# The yearly rate of Relative Thalamic Atrophy (yrRTA): a simple 2D/3D method for estimating deep gray matter atrophy in Multiple Sclerosis

**DOI:** 10.3389/fnagi.2014.00219

**Published:** 2014-08-26

**Authors:** Manuel Menéndez-González, José M. Salas-Pacheco, Oscar Arias-Carrión

**Affiliations:** ^1^Unidad de Neurología, Hospital Álvarez-BuyllaMieres, Spain; ^2^Departamento de Morfología y Biología Celular, Universidad de OviedoOviedo, Spain; ^3^Instituto de Neurociencias, Universidad de OviedoOviedo, Spain; ^4^Instituto de Investigación Científica, Universidad Juárez del Estado de DurangoDurango, México; ^5^Unidad de Trastornos del Movimiento y Sueño, Hospital General Dr. Manuel Gea González/Universidad Nacional Autónoma de MéxicoMexico City, Mexico; ^6^Unidad de Trastornos del Movimiento y Sueño, Hospital General Ajusco MedioMexico City, Mexico

**Keywords:** biomarker, gray matter, Multiple Sclerosis, atrophy, thalamus, clinically isolated syndrome, planimetry

## Abstract

Despite a strong correlation to outcome, the measurement of gray matter (GM) atrophy is not being used in daily clinical practice as a prognostic factor and monitor the effect of treatments in Multiple Sclerosis (MS). This is mainly because the volumetric methods available to date are sophisticated and difficult to implement for routine use in most hospitals. In addition, the meanings of raw results from volumetric studies on regions of interest are not always easy to understand. Thus, there is a huge need of a methodology suitable to be applied in daily clinical practice in order to estimate GM atrophy in a convenient and comprehensive way. Given the thalamus is the brain structure found to be more consistently implied in MS both in terms of extent of atrophy and in terms of prognostic value, we propose a solution based in this structure. In particular, we propose to compare the extent of thalamus atrophy with the extent of unspecific, global brain atrophy, represented by ventricular enlargement. We name this ratio the “yearly rate of Relative Thalamic Atrophy” (yrRTA). In this report we aim to describe the concept of yrRTA and the guidelines for computing it under 2D and 3D approaches and explain the rationale behind this method. We have also conducted a very short crossectional retrospective study to proof the concept of yrRTA. However, we do not seek to describe here the validity of this parameter since these researches are being conducted currently and results will be addressed in future publications.

## INTRODUCTION

Several studies have demonstrated that, on average, brain volume decreases by about 0.6–1% yearly in patients with Multiple Sclerosis (MS). Brain atrophy may involve both white matter (WM) and gray matter (GM; Miller et al., 2002). Although lower WM volume is associated with greater disability in MS, a study found WM volume on average in the normal range (Shiee et al., 2012). This paradoxical result might be explained by the presence of coexisting pathological processes, such as tissue damage and repair, that cause both atrophy and hypertrophy and that underlie the observed disability. GM damage is common and widespread in MS, especially in chronic MS but also in early stages, developing gradually following the appearance of inflammatory lesions (Miller et al., 2002). The underlying pathological correlates of GM damage in MS are different from WM damage. This probably reflects both inflammation-induced axonal loss followed by Wallerian degeneration and post-inflammatory neurodegeneration that may be partly due to failure of remyelination (Mühlau et al., 2013). However, one component of atrophy appears to be independent of focal lesions (Hulst and Geurts, 2011; Hoshi et al., 2013).

In contrast to GM atrophy, ventricular enlargement (VE) lacks specificity, representing a measure of global brain atrophy. It is well known that enlargement of lateral ventricles is a measure of unspecific global brain atrophy since it is strongly associated both with aging in healthy and with neurodegeneration. Almost any neurodegenerative disorder leads to some degree of VE, and so do some psychiatric conditions also. In MS, VE has been shown to be due to WM atrophy, deep brain atrophy and altered cerebrospinal fluid flow (Magnano et al., 2012). The exception here is the third ventricle where enlargement do seem to represent progression of the disease. Several studies, using well magnetic resonance imaging (MRI) well transcranial Doppler, showed that enlargement of the third ventricle correlates with motor deficits (Müller et al., 2013), disability (Kallmann et al., 2004), and cognition (Wollenweber et al., 2011; Müller et al., 2013) in MS.

Some authors found the pattern of atrophy is somewhat different in the different forms of MS: while subcortical damage is predominant in relapsing-remitting multiple sclerosis (RRMS; Pagani et al., 2005; Bergsland et al., 2012), cortical atrophy seems to be more important in the progressive forms (Pagani et al., 2005). However, other studies found lack of association with disease duration and disease category, suggesting that atrophy patterns may reflect different underlying mechanisms not distinguishable by conventional disease measures (Fisher et al., 2013).

In clinically isolated syndrome (CIS) changes in subcortical GM were reported (Henry et al., 2008). Indeed several studies found GM atrophy is associated with conversion to definite MS (Kalincik et al., 2012; Pérez-Miralles et al., 2013; Zivadinov et al., 2013), concluding that GM atrophy is a very good parameter to predict progression in CIS —and perhaps other forms of MS also—.

It is very clear that GM atrophy measurements correlate strongly with disability and cognitive impairment (more so than WM atrophy; Pagani et al., 2005; Riccitelli et al., 2011; Roosendaal et al., 2011; Shiee et al., 2012). The association of the distinct patterns of regional distribution of GM damage with cognition seems to depend on the clinical phenotypes: while in RRMS and SPMS patients there is a correspondence between presences of GM atrophy in several areas, this is not the case in PPMS patients (Riccitelli et al., 2011). Some brain structures also correlate better than others: for instance, high physical disability is associated with low thalamus and brainstem volumes but not with low cortical GM volume (Shiee et al., 2012).

Among all GM structures, the thalamus is the most consistently implied in MS. The paired thalamic nuclei on both sides of the third ventricle play major roles in cortical activation, relaying sensory and motor information to the higher cortical centers that influence cognition (Herrero et al., 2002). Thalamic involvement occurs within the first 5 years of MS onset, when most patients are still minimally disabled (Davies et al., 2005; Henry et al., 2008). Thalamus volumes are inversely correlated with lesion load in MS (Shiee et al., 2012) and thalamus atrophy (TA) is associated with a wide range of clinical manifestations including cognitive decline, motor deficits, disability, fatigue, painful syndromes, and ocular motility disturbances in patients with MS (Hulst and Geurts, 2011; Batista et al., 2012; Shiee et al., 2012; Minagar et al., 2013). TA is also associated with conversion from CIS to definite MS over 2 years (Vaneckova et al., 2013; Zivadinov et al., 2013). In addition, as TA leads to enlargement of the third ventricle, the selective specificity of the enlargement of the third ventricle in contrast to the unspecific enlargement of the lateral ventricles is an indirect evidence of the involvement of the thalamus in MS also.

## THE NEED

Magnetic resonance imaging plays an ever-expanding role in the evaluation of MS. Conventional MRI techniques, including lesion detection from T1- or T2-weighted images, have been the mainstay for monitoring disease activity, in which the use of gadolinium with T1-weighted images adds additional sensitivity and specificity for areas of acute inflammation. Advanced imaging methods including magnetization transfer, fluid attenuated inversion recovery, diffusion, magnetic resonance spectroscopy, functional MRI, volumetry, and nuclear imaging techniques have added to our understanding of the pathogenesis of MS and may provide methods to monitor therapies more sensitively in the future. However, these advanced methods are limited by their cost, availability, complexity, and lack of validation (Bakshi et al., 2005).

Despite a strong correlation to outcome, the measurements of GM atrophy is not being used in daily clinical practice as a prognostic factor and monitor the effect of treatments in MS yet. This is mainly because the volumetric methods available to date are sophisticated and difficult to implement for routine use in most hospitals. In addition, the meanings of raw results from volumetric studies on regions of interest are not always easy to understand.

Albeit ongoing clinical trials with anti-inflammatory, remyelinating, or neuroprotective therapies are implementing volumetric studies for measuring atrophy as a sensitive measure of the neurodegenerative component in MS, again these results will be difficult to translate to clinical practice. There is a lack of connection between practice in trials and practice under real clinical conditions and this has to be solved with a method able to link volumetry with clinically available neuroimaging techniques.

## THE PROPOSED SOLUTION

Given the thalamus is the brain structure found to be more consistently implied in MS both in terms of extent of atrophy and in terms of prognostic value, we propose a solution based in this structure. In particular, we propose to compare the extent of TA with the extent of unspecific, global brain atrophy, represented by VE. Thus, the yearly rate of Relative Thalamic Atrophy (yrRTA) compares the extent of TA with the extent of VE measured in two MRIs performed at two different time-points. Both the TA and the VE can be measured using 2D (areas of regions of interest on one MRI slice only) or 3D (volumes of regions of interest) methodology.

In this report we aim to describe the guidelines for computing the yrRTA. However, we do not seek to describe here the validity of this parameter since these researches are being conducted currently and results will be addressed in future publications.

### GUIDELINES

#### Measures in the 2D method

In the 2D method we select the T1 sequence and take the axial slide passing through the foramen of Monro/*interthalamic adhesion.*

**FIGURE 1:**
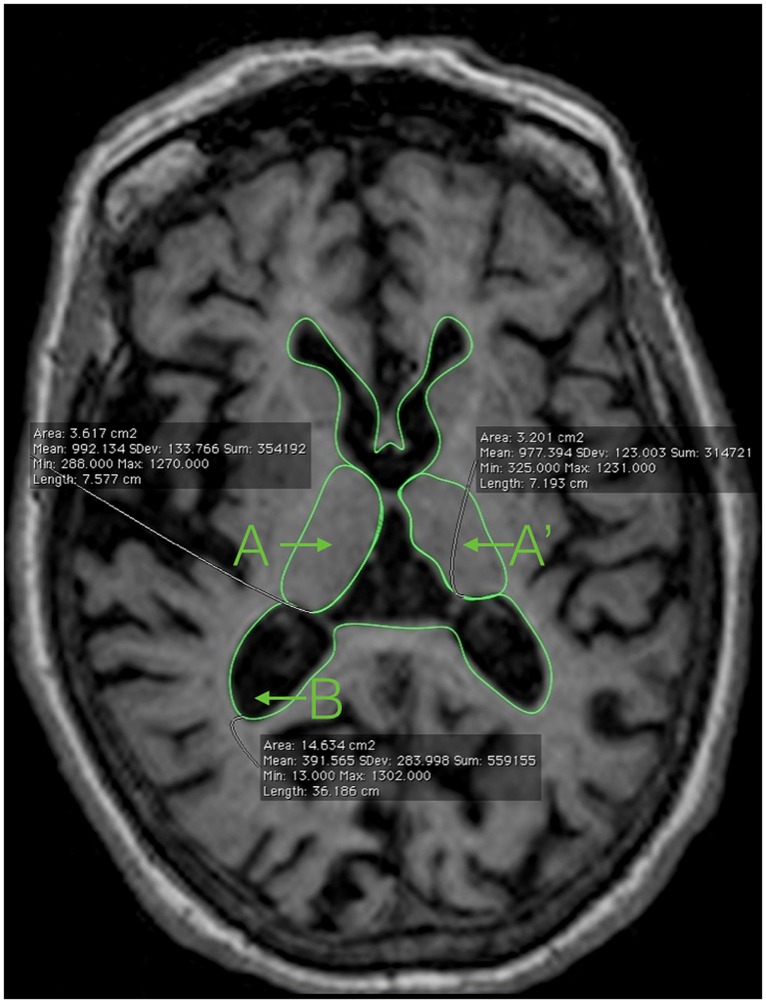
**Magnetic resonance imaging showing the areas needed to compute the 2D-yrRTA: A and A’: right and left thalamus; B: ventricular system including the lateral ventricles and the third ventricle**.

Using the tool for measuring areas available in most DICOM Viewer softwares, we just trace the next three regions of interest (**Figure 1**):

(1) The limits of each thalamus (Duvernoy, 1991; A, A′)

• Anterior: using the anterior commissure as a starting point, the thalamus can be identified just posterior to the commissure’s clearest coronal view. The anterior portion of the thalamus is narrow, resulting in an oblong shape in the coronal plane. It is this anterior section of the thalamus that forms the posterior boundary of the interventricular foramen.
• Lateral: throughout its course in the brain, the thalamus maintains its relationship with the internal capsule until the most posterior portion. The medial border of both the genu and the posterior limb of the internal capsule serve as the lateral border to the thalamus itself, thus separating the thalamus from the adjacent lentiform nucleus.
• Medial: the CSF of the third ventricle serves as the medial border of the thalamus. Where applicable, portions of the fornix may also serve as a medial border. It is important to note that left and right portions of the thalamus have to be traced individually, excluding the massa intermedia when present.
• Posterior: projecting beyond both the superior and inferior colliculi, the thalamus protrudes posteriorly until coming into contact with either the atrium of the lateral ventricle, the tail of the hippocampus, or both structures.

(2) The limits of the whole ventricular system, including the lateral ventricles, the third ventricle, and the posterior/occipital horn of the lateral ventricles (B). Tracing the limits of the ventricular system in 2D is very easy and do not require advanced anatomical knowledge (**Figure 1**).

#### Measures in the 3D method

There are different approaches and methodologies for performing volumetric studies, including manual, semi-automated, and fully automated methods (Miller et al., 2002). Any volumetric method is suitable for calculating the 3D-yrRTA. Usually, the tracer needs to know the limits and boundaries of the regions of interest. Here we need to find out the volume of each thalamus (A, A′), each lateral ventricles (C, C′), and the third ventricle (D).

The limits of the thalamus are as just described for the 2D method, and in addition we need to know the upper and lower borders of the thalamus (Duvernoy, 1991):

• Superiorly, the lateral ventricles bound the thalamus. As one moves from anterior to posterior, the thalamus loses its magnitude in the Y plane. This causes the coronal height of the thalamus to decrease, thus allowing the fornix to serve as the superior boundary in more posterior slices.
• Inferiorly, the zona incerta and its junction with the internal capsule serve as the inferior border of the thalamus, thus excluding the subthalamic nucleus, the substantia nigra, and the nucleus rubor.

The anatomical limits of the lateral ventricles and third ventricles are extensive, complex and difficult to resume here; we recommend consulting brain atlases for a review (i.e., Duvernoy, 1991). However, tracing the limits of these structures on MRI is quite simple given the distinct density of CSF in contrast to the density of any other brain structure.

#### Computing ratios

Thereafter, we can compute the following ratios using the formule described in **Table 1**.

**Table 1:** Formule for computing the yrTA, yrVE, and yrRTA under 2D and 3D methodologies.

	yrTA	yrVE	yrRTA
2D	(A1+A′1)-(A2+A′2) x 120**/**(#months between MRI studies)	(B2-B1) × 120**/**(#months between MRI studies)	(A1+A′1)-(A2+A′2) × 120**/**(B2-B1) × (#months between MRI studies)
3D	(A1+A′1)-(A2+A′2) × 120**/**(#months between MRI studies)	(C2+C’2+D2)-(C1+C’1+D1) × 120**/**(#months between MRI studies)	(A1+A′1)-(A2+A′2) × 120**/**(C2+C’2+D2)- (C1+C’1+D1) × (#months between MRI studies)

*Numbers 1 and 2 represent the first (1) and second (2) MRI studies.*

### CLINICAL INTERPRETATION OF RESULTS

Upcoming studies will allow us to set the normal rate of TA and the normal rate of VE. Then we will be able to classify the results of the yrTA and the yrVE as “low” or “high” in qualitative terms. Hereafter, the interpretation of results is easy to understand following the comments in **Table 2**. The expected risk of progression can be anticipated “*a priori*,” according to what we know regarding GM atrophy as marker of disability and progression in MS.

**Table 2:** Possible results of the yrTA, yrVE, and derived yrRTA in qualitative terms with the clinical interpretation.

yrTA	yrVE	yrRTA	Clinical interpretation
Low	Low	Normal	This is what we expect in a healthy subject or a patient without significant brain atrophy (no brain atrophy ongoing) – Low risk of progression
Low	High	Low	This is what we expect in someone whose brain is getting atrophied but the thalamus is not (atrophy ongoing but not due to deep GM atrophy) – Low risk of progression
High	Low	High	This is what we expect in someone whose thalamus is getting atrophied but other brain structures are not so significantly (atrophy ongoing due to deep GM atrophy mostly) – High risk of progression
High	High	Normal	This is what we expect in someone who has global brain atrophy including the thalamus (generalized atrophy ongoing) – High risk of progression

Although the yrRTA of patients who have global brain atrophy including TA might be similar to that of healthy subjects this situation is not a real challenge in clinical practice.

On one hand, patients with low yrRTA have “intact” deep GM, so they are expected to have good prognosis and low disability and cognitive impairment; therefore when clinicians face patients with low yrRTA a “conservative therapy” may be allowed. On the other hand, patients with high yrRTA are at high risk and an “aggressive therapy” may be needed. Patients with normal yrRTA but high yrTA and yrVE are probably those who are entering in a progressive form where neurodegeneration is becoming widespread and an “aggressive therapy” may also be needed. This scenario is different from that of someone who is in the latest stages of the disease, where the yrTA is no longer high and therefore the yrRTA is expected to be low or normal depending on the yrVE; but again, differentiating this situation from normal aging or low risk MS is not a real challenge since the clinical profile of these individuals is very different and easy to distinguish at first glance.

## PROOF-OF-CONCEPT

We conducted a very short crossectional retrospective study to proof the concept of yrRTA. It is not intended to prove the validity of the method, but to check the feasibility only. Thus, we do not provide here a full description of the methods nor performed advanced statistical analysis.

**FIGURE 2:**
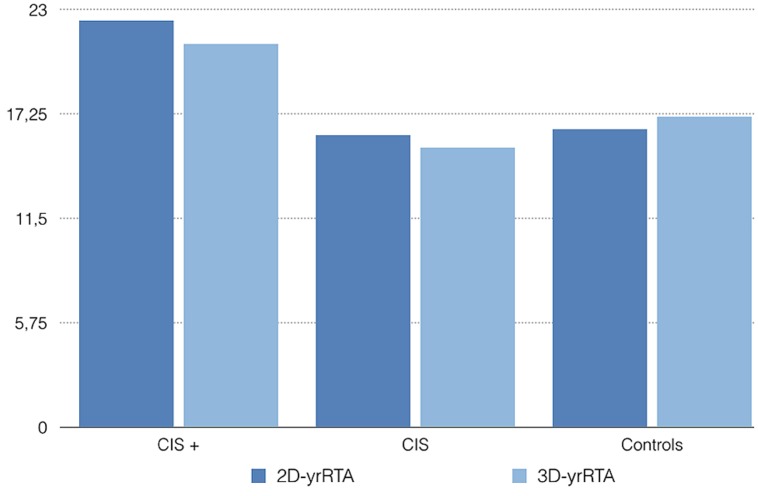
**Mean 2D- and 3D-yrRTA in patients with CIS who converted to MS over 24 months (CIS +), CIS who not converted (CIS) and healthy controls.** Values are merely illustrative -not informative-.

Experiments conform to the relevant regulatory standards in Europe and were approved by the Ethics Committee of Hospital Universitario Central de Asturias. Retrospectively, we took the baseline and 1 year (9–15 months) MRIs of five patients diagnosed with CIS who had converted to definite MS over 24 months and five patients diagnosed with CIS who had not converted to MS over 24 months. We also took the baseline and 1 year (9–15 months) MRIs of 5 age and gender matched controls. Then we traced the areas and volumes needed for the 2D- and 3D-yrRTA respectively using the software Osirix and computed the ratios as explained before. The mean 2D-yrRTA was 16.4 in controls, 16.1 in CIS who not progressed and 22.4 in CIS who progressed to MS. The 3D-yrRTA was 17.1 in controls, 15.4 in CIS who not progressed and 21.1 in CIS who progressed to MS (**Figure 2**).

As expected, both the 2D- and the 3D-yrRTA were higher in patients with CIS who progressed to MS than in controls or patients with CIS who not progressed. Obviously, with this small sample we cannot know if these differences are significative or not. Results of 2D- and 3D-yrRTA were similar in controls and CIS who not progressed. Results of 2D-yrRTA seem to be similar to those of 3D-yrRTA in all groups.

## ADVANTAGES AND LIMITATIONS OF THE yrRTA

From the clinician’s point of view, the yrRTA has the following advantages over other methods:

(1) Measurement and scoring of yrRTA is objective, providing a distinct advantage over visual techniques.
(2) Volumetric measurements require the use of special software, and much greater technical stringency in the acquisition of the MRI scans and are far more prone to a variety of measurement errors. Delineating the areas needed for calculating the 2D-yrRTA is fast and easy; little training is needed. This metric is very straightforward, therefore it can be implemented for daily clinical practice using basic neuroimaging facilities currently available in most hospitals with busy clinical settings.
(3) An additional advantage of using the yrRTA over absolute volumetric measures is that regional brain volumes are variable across individuals and need to be normalized by conversion to a ratio of the absolute volumes to intracranial volume, whereas the yrRTA has built-in normalization and thus avoids multiplicative errors inherent in using ratios of two quantitative variables.
(4) The same way, as aging affects lateral ventricles -independent of any pathology-, aging should be included as covariate in methods providing absolute volumes or scores. The yrRTA is an “intra-patient” ratio so it will not need cut-off scores adjusted by age because each subject serves as their own control.

On the other hand, the main limitation of the 2D-yrRTA is that scoring is based on measurements performed on a single slice, thereby providing a limited perspective of overall brain pathology. However, the correlation of the 2D- with the 3D-yrRTA will determine the validity of the 2D approach in this regard. It is also expected that other conditions affecting the ventricular morphology, such as hydrocephalus, can alter the interpretation of the yrRTA.

This paper is a methodological description only. Cut-off values have to be calculated for both the 2D- and the 3D-yrRTA and correlation between the 2D and 3D method has to be proven. Then, their use as a parameter for diagnosing MS in research and clinical practice has to be validated. Particularly, prospective studies are needed to assess the usefulness of the yrRTA as a parameter for predicting progression in the different forms of MS and its correlation with cognition and disability. It will be also interesting to explore the potential role of the yrRTA as a potential parameter helpful in the selection of treatments and in monitoring the efficacy.

## CONCLUSION

We report a new method for estimating deep GM atrophy in MS that is objective, comprehensive and easy to apply using clinically available neuroimaging. It may have some advantages over traditional volumetric methods that still need to be evaluated.

## Conflict of Interest Statement

The authors declare that the research was conducted in the absence of any commercial or financial relationships that could be construed as a potential conflict of interest.
